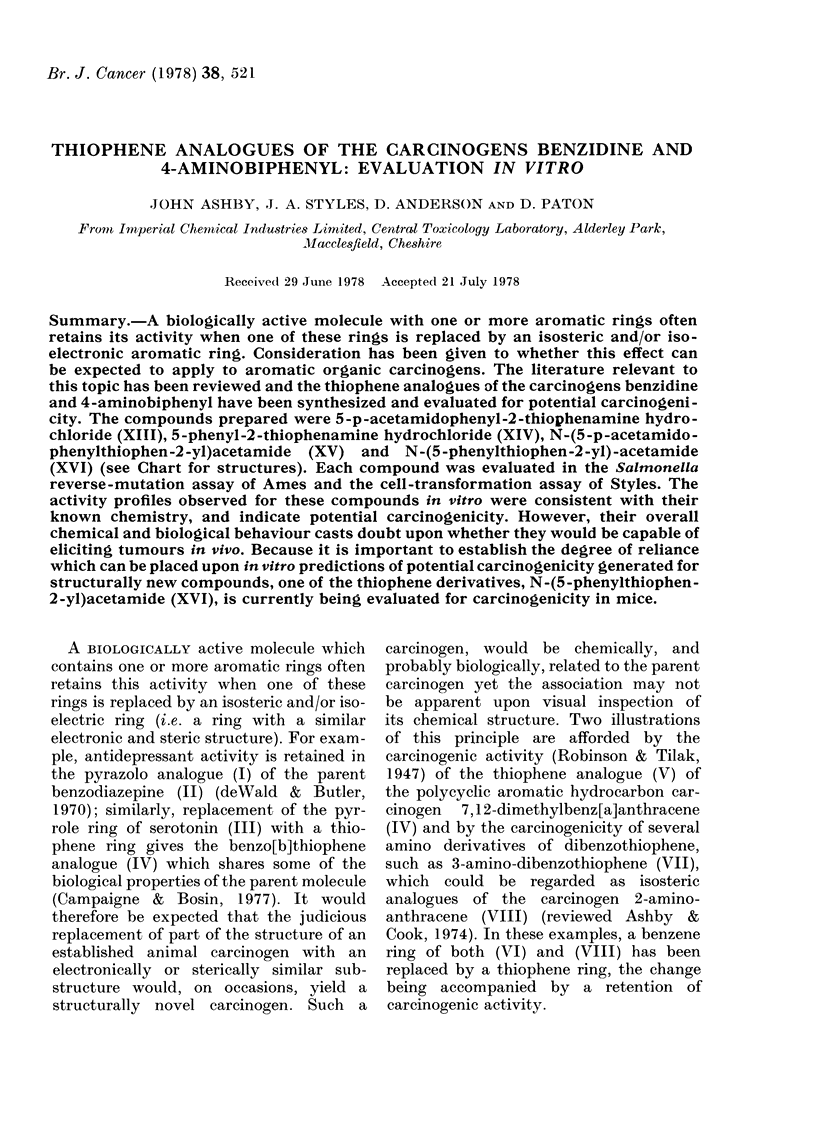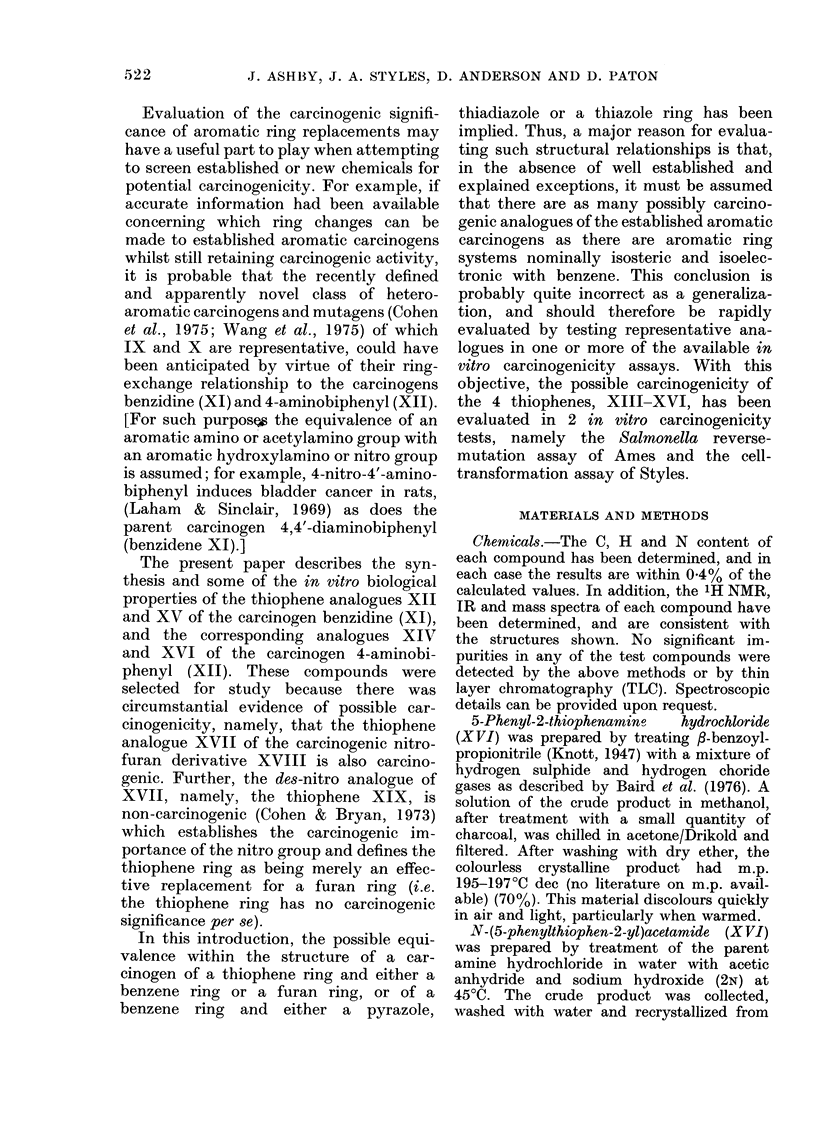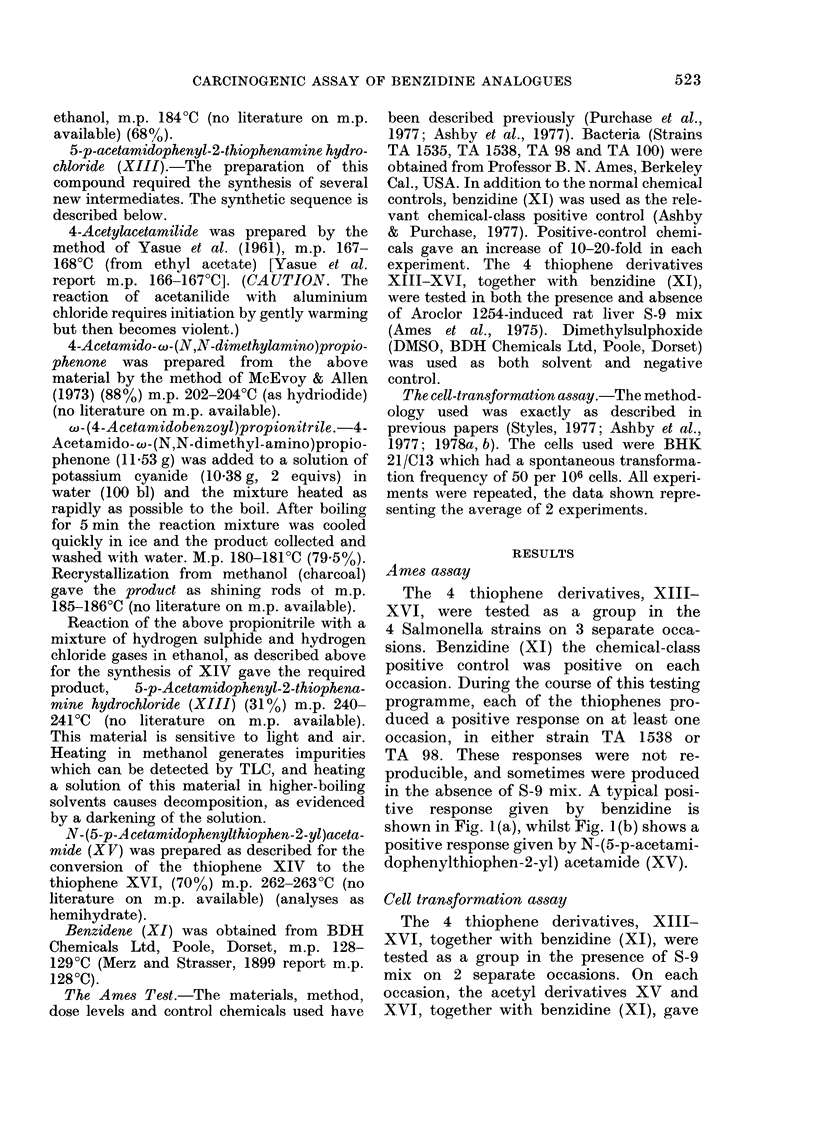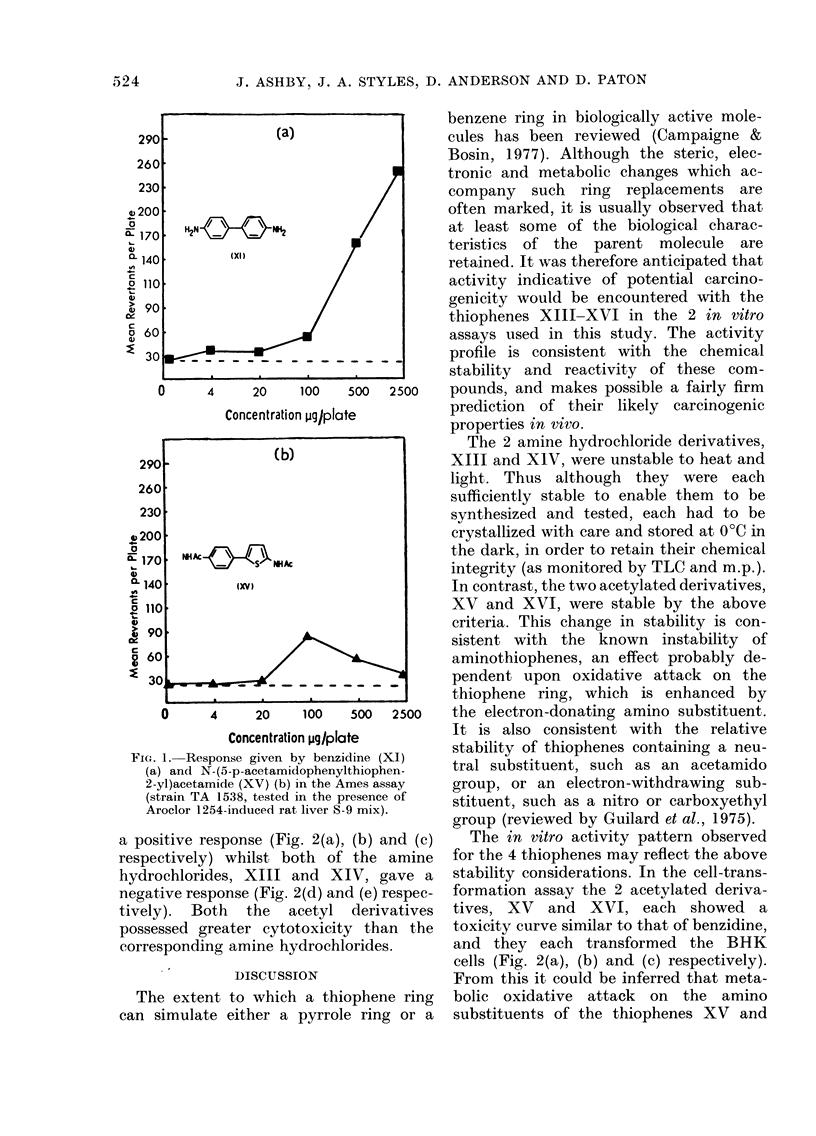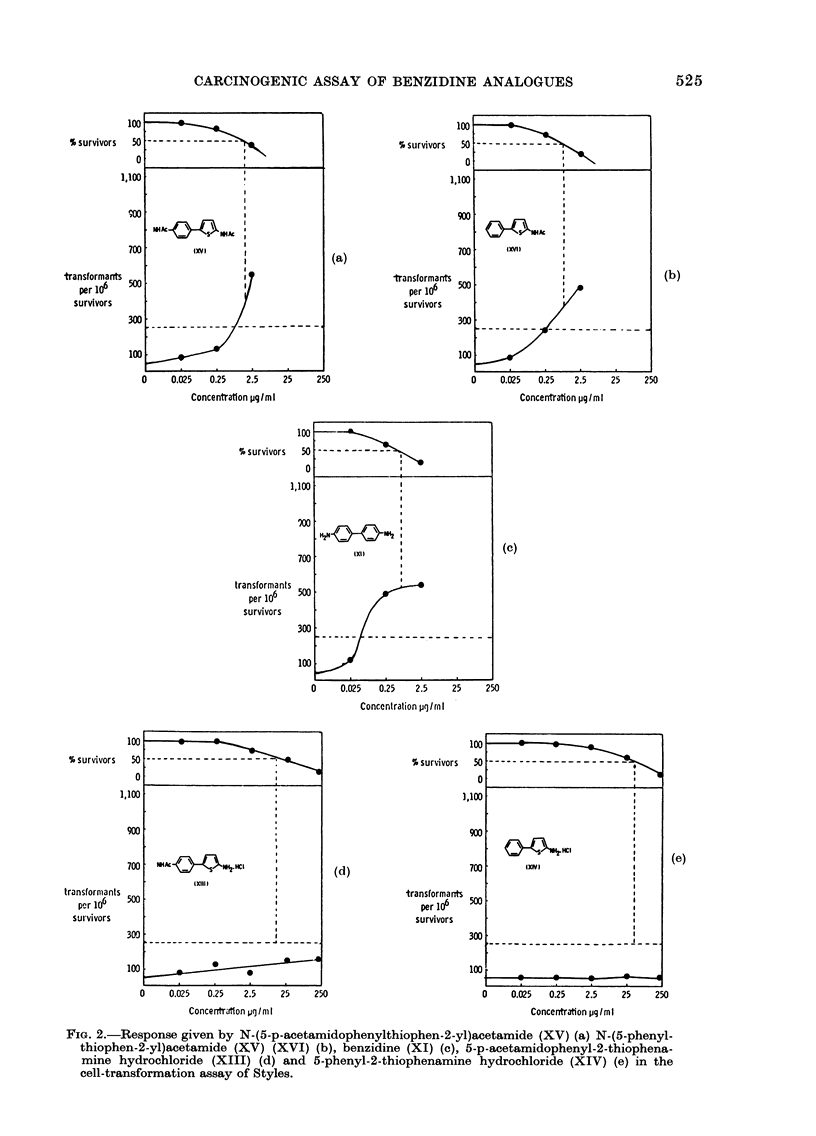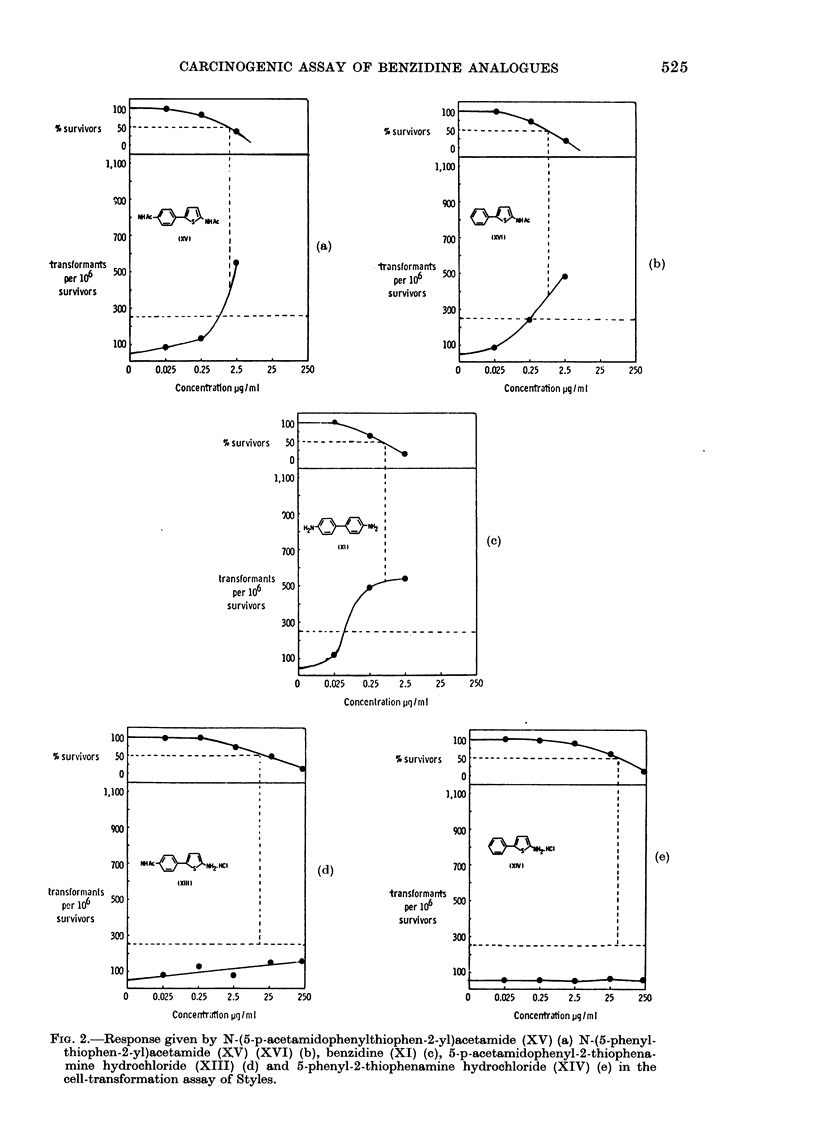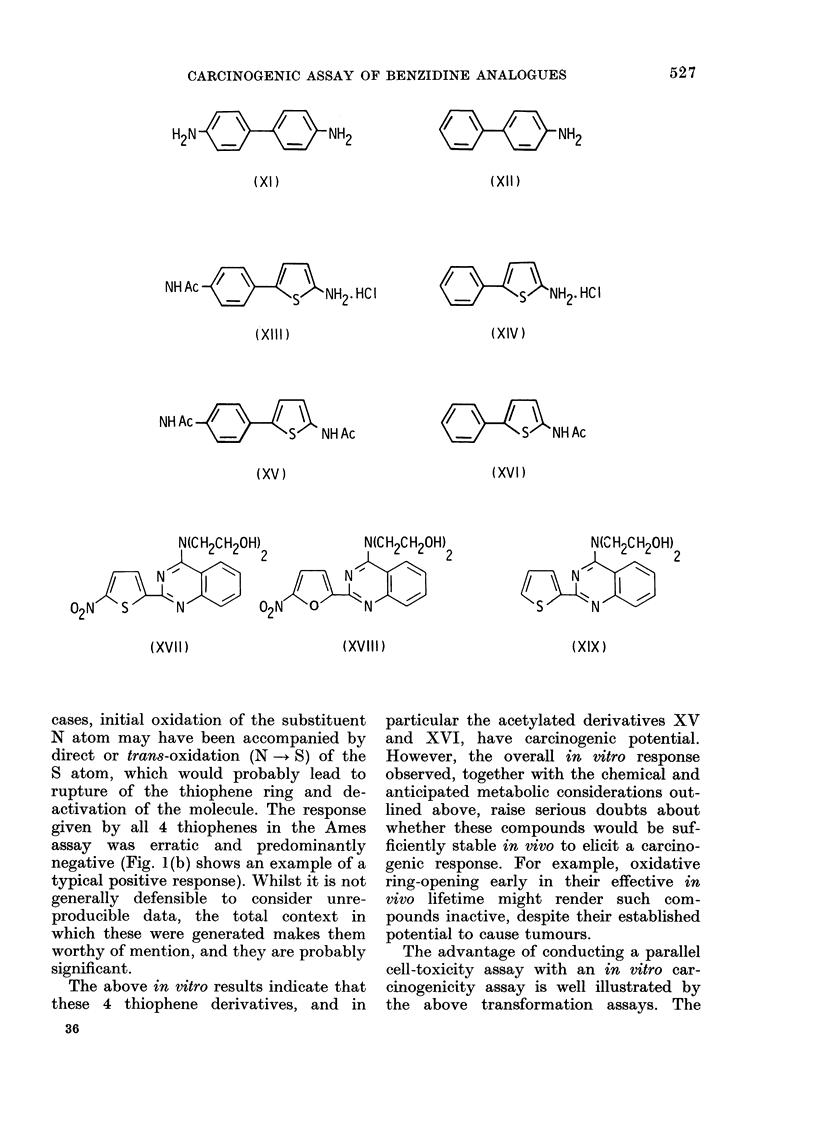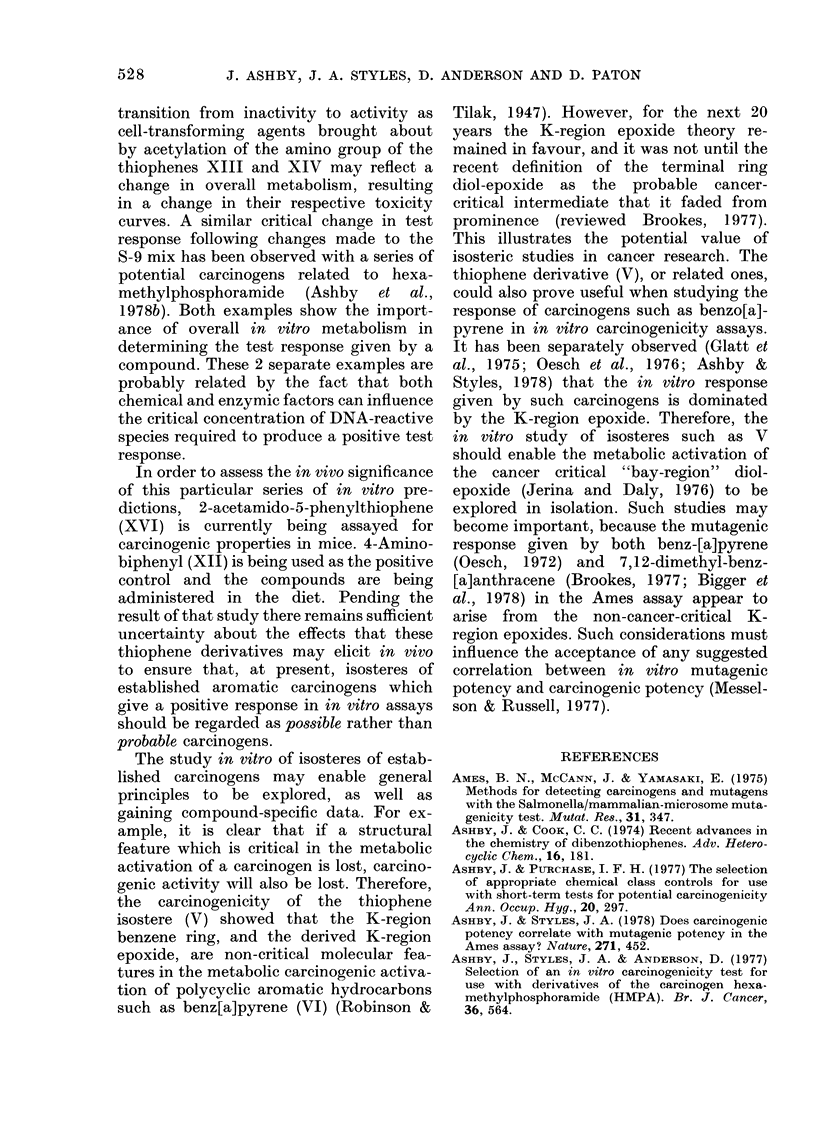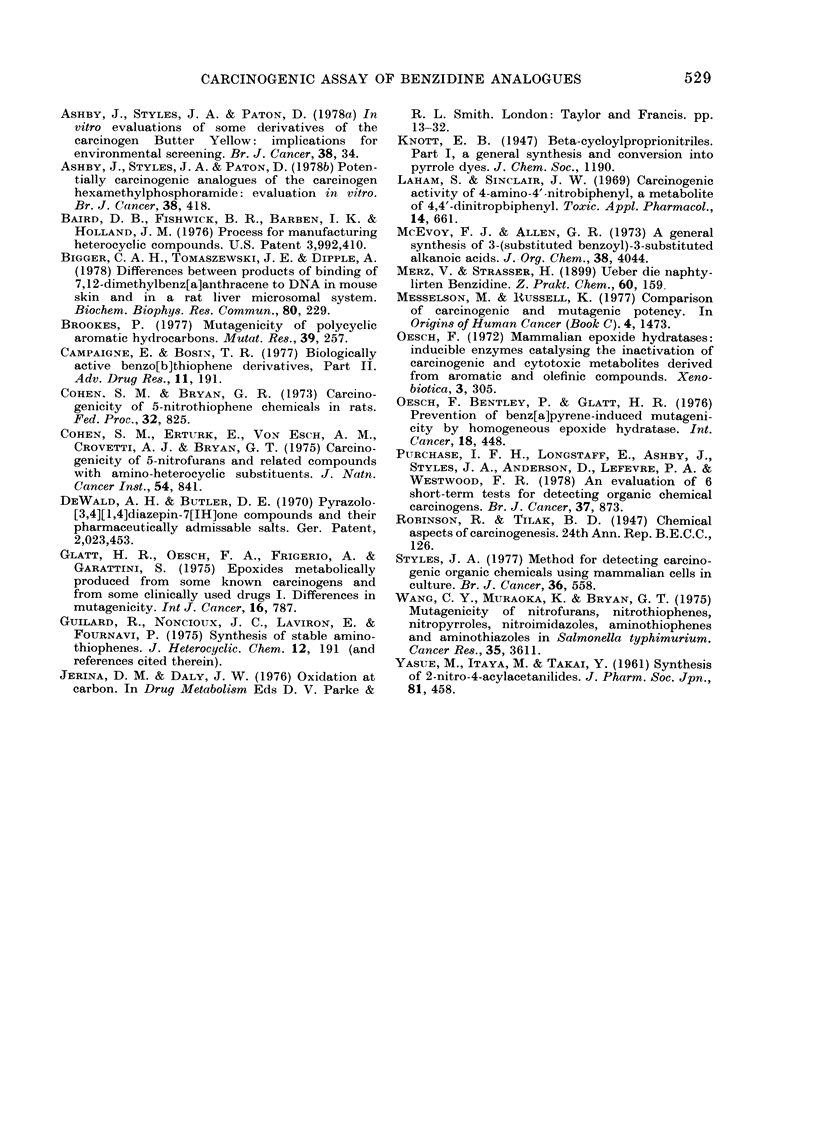# Thiophene analogues of the carcinogens benzidine and 4-aminobiphenyl: evaluation in vitro.

**DOI:** 10.1038/bjc.1978.239

**Published:** 1978-10

**Authors:** J. Ashby, J. A. Styles, D. Anderson, D. Paton

## Abstract

A biologically active molecule with one or more aromatic rings often retains its activity when one of these rings is replaced by an isosteric and/or isoelectronic aromatic ring. Consideration has been given to whether this effect can be expected to apply to aromatic organic carcinogens. The literature relevant to this topic has been reviewed and the thiophene analogues of the carcinogens benzidine and 4-aminobiphenyl have been synthesized and evaluated for potential carcinogenicity. The compounds prepared were 5-p-acetamidophenyl-2-thiophenamine hydrochloride (XIII), 5-phenyl-2-thiophenamine hydrochloride (XIV), N-(5-p-acetamido-phenylthiophen-2-yl)acetamide (XV) and N-(5-phenylthiophen-2-yl)-acetamide (XVI) (see Chart for structures). Each compound was evaluated in the Salmonella reverse-mutation assay of Ames and the cell-transformation assay of Styles. The activity profiles observed for these compounds in vitro were consistent with their known chemistry, and indicate potential carcinogenicity. However, their overall chemical and biological behaviour casts doubt upon whether they would be capable of eliciting tumours in vivo. Because it is important to establish the degree of reliance which can be placed upon in vitro predictions of potential carcinogenicity generated for structurally new compounds, one of the thiophene derivatives, N-(5-phenylthiophen-2-yl)acetamide ((XVI), is currently being evaluated for carcinogenicity in mice.


*Br. J. Cancer* (1978) **38**, 521

### THIOPHENE ANALOGUES OF THE CARCINOGENS BENZIDINE AND 4-AMINOBIPHENYL: EVALUATION *IN VITRO*

JOHN ASHBY, J. A. STYLES, D. ANDERSON AND D. PATON

*From Imperial Chemical Industries Limited, Central Toxicology Laboratory, Alderley Park, Macclesfield, Cheshire*

Received 29 June 1978 Accepted 21 July 1978

**Summary.—A biologically active molecule with one or more aromatic rings often retains its activity when one of these rings is replaced by an isosteric and/or iso-electronic aromatic ring. Consideration has been given to whether this effect can be expected to apply to aromatic organic carcinogens. The literature relevant to this topic has been reviewed and the thiophene analogues of the carcinogens benzidine and 4-aminobiphenyl have been synthesized and evaluated for potential carcinogenicity. The compounds prepared were 5-p-acetamidophenyl-2-thiophenamine hydrochloride (XIII), 5-phenyl-2-thiophenamine hydrochloride (XIV), N-(5-p-acetamidophenylthiophen-2-yl)acetamide (XV) and N-(5-phenylthiophen-2-yl)-acetamide (XVI) (see Chart for structures). Each compound was evaluated in the *Salmonella* reverse-mutation assay of Ames and the cell-transformation assay of Styles. The activity profiles observed for these compounds *in vitro* were consistent with their known chemistry, and indicate potential carcinogenicity. However, their overall chemical and biological behaviour casts doubt upon whether they would be capable of eliciting tumours *in vivo*. Because it is important to establish the degree of reliance which can be placed upon *in vitro* predictions of potential carcinogenicity generated for structurally new compounds, one of the thiophene derivatives, N-(5-phenylthiophen-2-yl)acetamide (XVI), is currently being evaluated for carcinogenicity in mice.**

A BIOLOGICALLY active molecule which contains one or more aromatic rings often retains this activity when one of these rings is replaced by an isosteric and/or iso-electric ring (*i.e.* a ring with a similar electronic and steric structure). For example, antidepressant activity is retained in the pyrazolo analogue (I) of the parent benzodiazepine (II) (deWald & Butler, 1970); similarly, replacement of the pyrrole ring of serotonin (III) with a thiophene ring gives the benzo[b]thiophene analogue (IV) which shares some of the biological properties of the parent molecule (Campaigne & Bosin, 1977). It would therefore be expected that the judicious replacement of part of the structure of an established animal carcinogen with an electronically or sterically similar sub-structure would, on occasions, yield a structurally novel carcinogen. Such a carcinogen, would be chemically, and probably biologically, related to the parent carcinogen yet the association may not be apparent upon visual inspection of its chemical structure. Two illustrations of this principle are afforded by the carcinogenic activity (Robinson & Tilak, 1947) of the thiophene analogue (V) of the polycyclic aromatic hydrocarbon carcinogen 7,12-dimethylbenz[a]anthracene (IV) and by the carcinogenicity of several amino derivatives of dibenzothiophene, such as 3-amino-dibenzothiophene (VII), which could be regarded as isosteric analogues of the carcinogen 2-amino-anthracene (VIII) (reviewed Ashby & Cook, 1974). In these examples, a benzene ring of both (VI) and (VIII) has been replaced by a thiophene ring, the change being accompanied by a retention of carcinogenic activity.

Evaluation of the carcinogenic significance of aromatic ring replacements may have a useful part to play when attempting to screen established or new chemicals for potential carcinogenicity. For example, if accurate information had been available concerning which ring changes can be made to established aromatic carcinogens whilst still retaining carcinogenic activity, it is probable that the recently defined and apparently novel class of heteroaromatic carcinogens and mutagens (Cohen *et al.*, 1975; Wang *et al.*, 1975) of which IX and X are representative, could have been anticipated by virtue of their ring-exchange relationship to the carcinogens benzidine (XI) and 4-aminobiphenyl (XII). [For such purposes the equivalence of an aromatic amino or acetylamino group with an aromatic hydroxylamino or nitro group is assumed; for example, 4-nitro-4′-aminobiphenyl induces bladder cancer in rats, (Laham & Sinclair, 1969) as does the parent carcinogen 4,4′-diaminobiphenyl (benzidene XI).]

The present paper describes the synthesis and some of the *in vitro* biological properties of the thiophene analogues XII and XV of the carcinogen benzidine (XI), and the corresponding analogues XIV and XVI of the carcinogen 4-aminobiphenyl (XII). These compounds were selected for study because there was circumstantial evidence of possible carcinogenicity, namely, that the thiophene analogue XVII of the carcinogenic nitrofuran derivative XVIII is also carcinogenic. Further, the *des*-nitro analogue of XVII, namely, the thiophene XIX, is non-carcinogenic (Cohen & Bryan, 1973) which establishes the carcinogenic importance of the nitro group and defines the thiophene ring as being merely an effective replacement for a furan ring (*i.e.* the thiophene ring has no carcinogenic significance *per se*).

In this introduction, the possible equivalence within the structure of a carcinogen of a thiophene ring and either a benzene ring or a furan ring, or of a benzene ring and either a pyrazole, thiadiazole or a thiazole ring has been implied. Thus, a major reason for evaluating such structural relationships is that, in the absence of well established and explained exceptions, it must be assumed that there are as many possibly carcinogenic analogues of the established aromatic carcinogens as there are aromatic ring systems nominally isosteric and isoelectronic with benzene. This conclusion is probably quite incorrect as a generalization, and should therefore be rapidly evaluated by testing representative analogues in one or more of the available *in vitro* carcinogenicity assays. With this objective, the possible carcinogenicity of the 4 thiophenes, XIII–XVI, has been evaluated in 2 *in vitro* carcinogenicity tests, namely the *Salmonella* reverse-mutation assay of Ames and the cell-transformation assay of Styles.

### MATERIALS AND METHODS

*Chemicals.*—The C, H and N content of each compound has been determined, and in each case the results are within 0·4% of the calculated values. In addition, the $^1$H NMR, IR and mass spectra of each compound have been determined, and are consistent with the structures shown. No significant impurities in any of the test compounds were detected by the above methods or by thin layer chromatography (TLC). Spectroscopic details can be provided upon request.

*5-Phenyl-2-thiophenamine hydrochloride (XVI)* was prepared by treating β-benzoylpropionitrile (Knott, 1947) with a mixture of hydrogen sulphide and hydrogen choride gases as described by Baird *et al.* (1976). A solution of the crude product in methanol, after treatment with a small quantity of charcoal, was chilled in acetone/Drikold and filtered. After washing with dry ether, the colourless crystalline product had m.p. 195–197°C dec (no literature on m.p. available) (70%). This material discolours quickly in air and light, particularly when warmed.

*N-(5-phenylthiophen-2-yl)acetamide (XVI)* was prepared by treatment of the parent amine hydrochloride in water with acetic anhydride and sodium hydroxide (2N) at 45°C. The crude product was collected, washed with water and recrystallized from ethanol, m.p. 184°C (no literature on m.p. available) (68%).

*5-p-acetamidophenyl-2-thiophenamine hydrochloride (XIII)*.—The preparation of this compound required the synthesis of several new intermediates. The synthetic sequence is described below.

*4-Acetylacetamilide* was prepared by the method of Yasue *et al.* (1961), m.p. 167–168°C (from ethyl acetate) [Yasue *et al.* report m.p. 166–167°C]. (*CAUTION*. The reaction of acetanilide with aluminium chloride requires initiation by gently warming but then becomes violent.)

*4-Acetamido-ω-(N,N-dimethylamino)propiophenone* was prepared from the above material by the method of McEvoy & Allen (1973) (88%) m.p. 202–204°C (as hydriodide) (no literature on m.p. available).

*ω-(4-Acetamidobenzoyl)propionitrile*.—4-Acetamido-ω-(N,N-dimethyl-amino)propiophenone (11·53 g) was added to a solution of potassium cyanide (10·38 g, 2 equivs) in water (100 bl) and the mixture heated as rapidly as possible to the boil. After boiling for 5 min the reaction mixture was cooled quickly in ice and the product collected and washed with water. M.p. 180–181°C (79·5%). Recrystallization from methanol (charcoal) gave the *product* as shining rods ot m.p. 185–186°C (no literature on m.p. available).

Reaction of the above propionitrile with a mixture of hydrogen sulphide and hydrogen chloride gases in ethanol, as described above for the synthesis of XIV gave the required product, *5-p-Acetamidophenyl-2-thiophenamine hydrochloride (XIII)* (31%) m.p. 240–241°C (no literature on m.p. available). This material is sensitive to light and air. Heating in methanol generates impurities which can be detected by TLC, and heating a solution of this material in higher-boiling solvents causes decomposition, as evidenced by a darkening of the solution.

*N-(5-p-Acetamidophenylthiophen-2-yl)acetamide (XV)* was prepared as described for the conversion of the thiophene XIV to the thiophene XVI, (70%) m.p. 262–263°C (no literature on m.p. available) (analyses as hemihydrate).

*Benzidene (XI)* was obtained from BDH Chemicals Ltd, Poole, Dorset, m.p. 128–129°C (Merz and Strasser, 1899 report m.p. 128°C).

*The Ames Test*.—The materials, method, dose levels and control chemicals used have been described previously (Purchase *et al.*, 1977; Ashby *et al.*, 1977). Bacteria (Strains TA 1535, TA 1538, TA 98 and TA 100) were obtained from Professor B. N. Ames, Berkeley Cal., USA. In addition to the normal chemical controls, benzidine (XI) was used as the relevant chemical-class positive control (Ashby & Purchase, 1977). Positive-control chemicals gave an increase of 10–20-fold in each experiment. The 4 thiophene derivatives XIII–XVI, together with benzidine (XI), were tested in both the presence and absence of Aroclor 1254-induced rat liver S-9 mix (Ames *et al.*, 1975). Dimethylsulphoxide (DMSO, BDH Chemicals Ltd, Poole, Dorset) was used as both solvent and negative control.

*The cell-transformation assay*.—The methodology used was exactly as described in previous papers (Styles, 1977; Ashby *et al.*, 1977; 1978*a*, *b*). The cells used were BHK 21/C13 which had a spontaneous transformation frequency of 50 per $10^6$ cells. All experiments were repeated, the data shown representing the average of 2 experiments.

### RESULTS

### *Ames assay*

The 4 thiophene derivatives, XIII–XVI, were tested as a group in the 4 Salmonella strains on 3 separate occasions. Benzidine (XI) the chemical-class positive control was positive on each occasion. During the course of this testing programme, each of the thiophenes produced a positive response on at least one occasion, in either strain TA 1538 or TA 98. These responses were not reproducible, and sometimes were produced in the absence of S-9 mix. A typical positive response given by benzidine is shown in Fig. 1(a), whilst Fig. 1(b) shows a positive response given by N-(5-p-acetamidophenylthiophen-2-yl) acetamide (XV).

### *Cell transformation assay*

The 4 thiophene derivatives, XIII–XVI, together with benzidine (XI), were tested as a group in the presence of S-9 mix on 2 separate occasions. On each occasion, the acetyl derivatives XV and XVI, together with benzidine (XI), gave

FIG. 1.—Response given by benzidine (XI) (a) and N-(5-p-acetamidophenylthiophen-2-yl)acetamide (XV) (b) in the Ames assay (strain TA 1538, tested in the presence of Aroclor 1254-induced rat liver S-9 mix).

a positive response (Fig. 2(a), (b) and (c) respectively) whilst both of the amine hydrochlorides, XIII and XIV, gave a negative response (Fig. 2(d) and (e) respectively). Both the acetyl derivatives possessed greater cytotoxicity than the corresponding amine hydrochlorides.

### DISCUSSION

The extent to which a thiophene ring can simulate either a pyrrole ring or a benzene ring in biologically active molecules has been reviewed (Campaigne & Bosin, 1977). Although the steric, electronic and metabolic changes which accompany such ring replacements are often marked, it is usually observed that at least some of the biological characteristics of the parent molecule are retained. It was therefore anticipated that activity indicative of potential carcinogenicity would be encountered with the thiophenes XIII–XVI in the 2 *in vitro* assays used in this study. The activity profile is consistent with the chemical stability and reactivity of these compounds, and makes possible a fairly firm prediction of their likely carcinogenic properties *in vivo*.

The 2 amine hydrochloride derivatives, XIII and XIV, were unstable to heat and light. Thus although they were each sufficiently stable to enable them to be synthesized and tested, each had to be crystallized with care and stored at 0°C in the dark, in order to retain their chemical integrity (as monitored by TLC and m.p.). In contrast, the two acetylated derivatives, XV and XVI, were stable by the above criteria. This change in stability is consistent with the known instability of aminothiophenes, an effect probably dependent upon oxidative attack on the thiophene ring, which is enhanced by the electron-donating amino substituent. It is also consistent with the relative stability of thiophenes containing a neutral substituent, such as an acetamido group, or an electron-withdrawing substituent, such as a nitro or carboxyethyl group (reviewed by Guilard *et al.*, 1975).

The *in vitro* activity pattern observed for the 4 thiophenes may reflect the above stability considerations. In the cell-transformation assay the 2 acetylated derivatives, XV and XVI, each showed a toxicity curve similar to that of benzidine, and they each transformed the BHK cells (Fig. 2(a), (b) and (c) respectively). From this it could be inferred that metabolic oxidative attack on the amino substituents of the thiophenes XV and

FIG. 2.—Response given by N-(5-p-acetamidophenylthiophen-2-yl)acetamide (XV) (a) N-(5-phenyl-thiophen-2-yl)acetamide (XV) (XVI) (b), benzidine (XI) (c), 5-p-acetamidophenyl-2-thiophenamine hydrochloride (XIII) (d) and 5-phenyl-2-thiophenamine hydrochloride (XIV) (e) in the cell-transformation assay of Styles.

FIG. 2.—Response given by N-(5-p-acetamidophenylthiophen-2-yl)acetamide (XV) (a) N-(5-phenylthiophen-2-yl)acetamide (XV) (XVI) (b), benzidine (XI) (c), 5-p-acetamidophenyl-2-thiophenamine hydrochloride (XIII) (d) and 5-phenyl-2-thiophenamine hydrochloride (XIV) (e) in the cell-transformation assay of Styles.

(XI)

(XII)

(XIII)

(XIV)

(XV)

(XVI)

(XVII)

(XVIII)

(XIX)

cases, initial oxidation of the substituent N atom may have been accompanied by direct or *trans*-oxidation (N → S) of the S atom, which would probably lead to rupture of the thiophene ring and deactivation of the molecule. The response given by all 4 thiophenes in the Ames assay was erratic and predominantly negative (Fig. 1(b) shows an example of a typical positive response). Whilst it is not generally defensible to consider unreproducible data, the total context in which these were generated makes them worthy of mention, and they are probably significant.

The above *in vitro* results indicate that these 4 thiophene derivatives, and in particular the acetylated derivatives XV and XVI, have carcinogenic potential. However, the overall *in vitro* response observed, together with the chemical and anticipated metabolic considerations outlined above, raise serious doubts about whether these compounds would be sufficiently stable *in vivo* to elicit a carcinogenic response. For example, oxidative ring-opening early in their effective *in vivo* lifetime might render such compounds inactive, despite their established potential to cause tumours.

The advantage of conducting a parallel cell-toxicity assay with an *in vitro* carcinogenicity assay is well illustrated by the above transformation assays. The transition from inactivity to activity as cell-transforming agents brought about by acetylation of the amino group of the thiophenes XIII and XIV may reflect a change in overall metabolism, resulting in a change in their respective toxicity curves. A similar critical change in test response following changes made to the S-9 mix has been observed with a series of potential carcinogens related to hexamethylphosphoramide (Ashby *et al.*, 1978*b*). Both examples show the importance of overall *in vitro* metabolism in determining the test response given by a compound. These 2 separate examples are probably related by the fact that both chemical and enzymic factors can influence the critical concentration of DNA-reactive species required to produce a positive test response.

In order to assess the *in vivo* significance of this particular series of *in vitro* predictions, 2-acetamido-5-phenylthiophene (XVI) is currently being assayed for carcinogenic properties in mice. 4-Aminobiphenyl (XII) is being used as the positive control and the compounds are being administered in the diet. Pending the result of that study there remains sufficient uncertainty about the effects that these thiophene derivatives may elicit *in vivo* to ensure that, at present, isosteres of established aromatic carcinogens which give a positive response in *in vitro* assays should be regarded as *possible* rather than *probable* carcinogens.

The study *in vitro* of isosteres of established carcinogens may enable general principles to be explored, as well as gaining compound-specific data. For example, it is clear that if a structural feature which is critical in the metabolic activation of a carcinogen is lost, carcinogenic activity will also be lost. Therefore, the carcinogenicity of the thiophene isostere (V) showed that the K-region benzene ring, and the derived K-region epoxide, are non-critical molecular features in the metabolic carcinogenic activation of polycyclic aromatic hydrocarbons such as benz[a]pyrene (VI) (Robinson & Tilak, 1947). However, for the next 20 years the K-region epoxide theory remained in favour, and it was not until the recent definition of the terminal ring diol-epoxide as the probable cancer-critical intermediate that it faded from prominence (reviewed Brookes, 1977). This illustrates the potential value of isosteric studies in cancer research. The thiophene derivative (V), or related ones, could also prove useful when studying the response of carcinogens such as benzo[a]-pyrene in *in vitro* carcinogenicity assays. It has been separately observed (Glatt *et al.*, 1975; Oesch *et al.*, 1976; Ashby & Styles, 1978) that the *in vitro* response given by such carcinogens is dominated by the K-region epoxide. Therefore, the *in vitro* study of isosteres such as V should enable the metabolic activation of the cancer critical "bay-region" diol-epoxide (Jerina and Daly, 1976) to be explored in isolation. Such studies may become important, because the mutagenic response given by both benz-[a]pyrene (Oesch, 1972) and 7,12-dimethyl-benz-[a]anthracene (Brookes, 1977; Bigger *et al.*, 1978) in the Ames assay appear to arise from the non-cancer-critical K-region epoxides. Such considerations must influence the acceptance of any suggested correlation between *in vitro* mutagenic potency and carcinogenic potency (Messelson & Russell, 1977).

### REFERENCES

AMES, B. N., MCCANN, J. & YAMASAKI, E. (1975) Methods for detecting carcinogens and mutagens with the Salmonella/mammalian-microsome mutagenicity test. *Mutat. Res.*, **31**, 347.

ASHBY, J. & COOK, C. C. (1974) Recent advances in the chemistry of dibenzothiophenes. *Adv. Heterocyclic Chem.*, **16**, 181.

ASHBY, J. & PURCHASE, I. F. H. (1977) The selection of appropriate chemical class controls for use with short-term tests for potential carcinogenicity *Ann. Occup. Hyg.*, **20**, 297.

ASHBY, J. & STYLES, J. A. (1978) Does carcinogenic potency correlate with mutagenic potency in the Ames assay? *Nature*, **271**, 452.

ASHBY, J., STYLES, J. A. & ANDERSON, D. (1977) Selection of an *in vitro* carcinogenicity test for use with derivatives of the carcinogen hexamethylphosphoramide (HMPA). *Br. J. Cancer*, **36**, 564.

Ashby, J., Styles, J. A. & Paton, D. (1978*a*) *In vitro* evaluations of some derivatives of the carcinogen Butter Yellow: implications for environmental screening. *Br. J. Cancer*, **38**, 34.

Ashby, J., Styles, J. A. & Paton, D. (1978*b*) Potentially carcinogenic analogues of the carcinogen hexamethylphosphoramide: evaluation *in vitro*. *Br. J. Cancer*, **38**, 418.

Baird, D. B., Fishwick, B. R., Barben, I. K. & Holland, J. M. (1976) Process for manufacturing heterocyclic compounds. U.S. Patent 3,992,410.

Bigger, C. A. H., Tomaszewski, J. E. & Dipple, A. (1978) Differences between products of binding of 7,12-dimethylbenz[a]anthracene to DNA in mouse skin and in a rat liver microsomal system. *Biochem. Biophys. Res. Commun.*, **80**, 229.

Brookes, P. (1977) Mutagenicity of polycyclic aromatic hydrocarbons. *Mutat. Res.*, **39**, 257.

Campaigne, E. & Bosin, T. R. (1977) Biologically active benzo[b]thiophene derivatives, Part II. *Adv. Drug Res.*, **11**, 191.

Cohen. S. M. & Bryan, G. R. (1973) Carcinogenicity of 5-nitrothiophene chemicals in rats. *Fed. Proc.*, **32**, 825.

Cohen, S. M., Erturk, E., Von Esch, A. M., Crovetti, A. J. & Bryan, G. T. (1975) Carcinogenicity of 5-nitrofurans and related compounds with amino-heterocyclic substituents. *J. Natn. Cancer Inst.*, **54**, 841.

DeWald, A. H. & Butler, D. E. (1970) Pyrazolo-[3,4][1,4]diazepin-7[IH]one compounds and their pharmaceutically admissable salts. Ger. Patent, 2,023,453.

Glatt, H. R., Oesch, F. A., Frigerio, A. & Garattini, S. (1975) Epoxides metabolically produced from some known carcinogens and from some clinically used drugs I. Differences in mutagenicity. *Int J. Cancer*, **16**, 787.

Guilard, R., Noncioux, J. C., Laviron, E. & Fournavi, P. (1975) Synthesis of stable aminothiophenes. *J. Heterocyclic. Chem.* **12**, 191 (and references cited therein).

Jerina, D. M. & Daly, J. W. (1976) Oxidation at carbon. In *Drug Metabolism* Eds D. V. Parke & R. L. Smith. London: Taylor and Francis. pp. 13–32.

Knott, E. B. (1947) Beta-cycloylproprionitriles. Part I, a general synthesis and conversion into pyrrole dyes. *J. Chem. Soc.*, 1190.

Laham, S. & Sinclair, J. W. (1969) Carcinogenic activity of 4-amino-4′-nitrobiphenyl, a metabolite of 4,4′-dinitropbiphenyl. *Toxic. Appl. Pharmacol.*, **14**, 661.

McEvoy, F. J. & Allen, G. R. (1973) A general synthesis of 3-(substituted benzoyl)-3-substituted alkanoic acids. *J. Org. Chem.*, **38**, 4044.

Merz, V. & Strasser, H. (1899) Ueber die naphtylirten Benzidine. *Z. Prakt. Chem.*, **60**, 159.

Messelson, M. & Russell, K. (1977) Comparison of carcinogenic and mutagenic potency. In *Origins of Human Cancer* (*Book C*). **4**, 1473.

Oesch, F. (1972) Mammalian epoxide hydratases: inducible enzymes catalysing the inactivation of carcinogenic and cytotoxic metabolites derived from aromatic and olefinic compounds. *Xenobiotica*, **3**, 305.

Oesch, F. Bentley, P. & Glatt, H. R. (1976) Prevention of benz[a]pyrene-induced mutagenicity by homogeneous epoxide hydratase. *Int. Cancer*, **18**, 448.

Purchase, I. F. H., Longstaff, E., Ashby, J., Styles, J. A., Anderson, D., Lefevre, P. A. & Westwood, F. R. (1978) An evaluation of 6 short-term tests for detecting organic chemical carcinogens. *Br. J. Cancer*, **37**, 873.

Robinson, R. & Tilak, B. D. (1947) Chemical aspects of carcinogenesis. 24th Ann. Rep. B.E.C.C., 126.

Styles, J. A. (1977) Method for detecting carcinogenic organic chemicals using mammalian cells in culture. *Br. J. Cancer*, **36**, 558.

Wang, C. Y., Muraoka, K. & Bryan, G. T. (1975) Mutagenicity of nitrofurans, nitrothiophenes, nitropyrroles, nitroimidazoles, aminothiophenes and aminothiazoles in *Salmonella typhimurium*. *Cancer Res.*, **35**, 3611.

Yasue, M., Itaya, M. & Takai, Y. (1961) Synthesis of 2-nitro-4-acylacetanilides. *J. Pharm. Soc. Jpn.*, **81**, 458.